# Research on coupling evacuation of escalator and staircase in fire scenario

**DOI:** 10.1371/journal.pone.0314455

**Published:** 2025-02-04

**Authors:** Chunhua Zhang, Xin Wu, Hai Shen

**Affiliations:** 1 College of Safety Science and Engineering, Liaoning Technical University, Fuxin, China; 2 Key Laboratory of Mine Thermodynamic Disasters and Control of Ministry of Education (Liaoning Technical University), Fuxin, China; 3 Jingneng (Xilin Gol) Mining Co. LTD, Abaga Banner of Xilin Gol League Inner Mongolia, China; The University of Edinburgh, UNITED KINGDOM OF GREAT BRITAIN AND NORTHERN IRELAND

## Abstract

In order to improve mall evacuation efficiency, Pyrosim software and Anylogic software are used to study the coupled evacuation of mall personnel using escalators and stairs in a fire scenario. The available safe evacuation time of each evacuation exit was explored by analyzing the smoke transport, temperature, CO concentration and visibility of each floor of the mall under different fire source locations when the fire shutter was lowered to 1.8m from the ground. Then, the evacuation model of mall personnel under fire scenario was built by using Anylogic to compare and analyze the evacuation time of mall personnel using escalators coupled with stairs and stairs only, and analyze the evacuation time of personnel using escalators coupled with stairs when the fire shutter at the escalator is down to 1.8m from the ground and when the fire shutter is not down. The study shows that when the fire source is located on the top floor of the mall, the smoke does not affect the evacuation of people on other floors during the simulation time. Under the fire scenario, the escalator-coupled staircase evacuation can shorten the maximum evacuation time by 23.37% compared with the staircase only. The fire shutter descends to 1.8m from the ground and the evacuation efficiency is 6.41% higher than when the fire shutter is not descended. This research contributes to the enhancement of mall safety and has practical implications for future emergency management strategies in public spaces.

## Introduction

The increasing prevalence of atrium structures in buildings, particularly shopping malls, has become a significant trend in response to societal development [1]. Atrium structures possess unique characteristics, such as interconnected floors. In the event of a fire, high-temperature smoke rapidly spreads across each floor, posing risks of asphyxiation or disorientation to evacuees, thus significantly impeding their escape efforts [2]. Furthermore, pedestrians in everyday life are less familiar with evacuation stairs in atrium malls and tend to predominantly rely on escalators or elevators for vertical movement. Consequently, during a fire emergency, the immediate reaction of personnel would be to gravitate towards the nearest escalator [3]. Therefore, this study conducts fire simulations using Pyrosim software and considers escalators as supplementary evacuation stairs within a predetermined safe timeframe. The objective is to augment evacuation passages and enhance the efficiency of personnel evacuation.

The utilization of escalators for evacuation in the event of fire incidents in buildings has been restricted by fire regulations. However, there are exceptions made for escalators in subway stations that adhere to specified standards. Previous research conducted by domestic and international scholars has shed light on the potential role of escalators in emergency evacuation scenarios. Huang [4] was the first to propose the potential function of escalators in ensuring safety during evacuations. Naoko et al. [5] conducted evacuation experiments utilizing escalators of varying heights and lengths, examining both stationary and upward movement modes to determine the evacuation speed of pedestrians utilizing escalators. The findings of their study provided a foundation for subsequent researchers in assessing the movement speed of escalators during evacuations. Hiroyuki et al. [6] performed numerical simulations on a subway station that implemented escalators for evacuation, comparing their results to scenarios where only staircases were utilized. Their simulations revealed that the evacuation completion time was approximately halved when escalators were incorporated. Tang [7], using the Pathfinder software, conducted simulations to compare the evacuation times of a commercial complex employing purely staircases versus utilizing escalators for evacuation. The study demonstrated that the inclusion of escalators resulted in a reduction in the evacuation time of individuals. Ma et al. [8] utilized the Pathfinder software to optimize the coupling of escalators and staircases as dual evacuation channels. Their findings indicated that the evacuation time in the coupled evacuation mode was 16.24% shorter than that of a singular staircase evacuation mode. Xie et al. [9] concluded that modifying the operation of escalators in subway stations, transitioning them from descending mode to ascending mode, can enhance evacuation efficiency to a certain degree.

The current research on escalators as evacuation staircases has achieved results, but the following problems still exist: The previous researchers regarded escalators as an emergency evacuation measure and regarded them as evacuation paths during the whole evacuation process, without considering the effect of fire on escalator evacuation. Since the movement and spread of smoke during a fire can significantly affect the evacuation process of a building [10, 11]. Wang et al. [12] proposed a method to obtain an optimal staged evacuation strategy by combining the dynamic movement of smoke and the evacuation process. Lotfi et al. [13] utilized the results of numerical simulation of a fire to find a safe evacuation exit. Yan et al. [14] simulated the necessary parameters of the fire scenario, such as temperature, visibility, and CO concentration, to obtain the available safe evacuation time., thus obtaining the available safe evacuation time and obtaining that the elevator can be used for evacuation when the fire source is far away from the elevator. Cui et al. [15] performed numerical fire simulation of a school building using Pyrosim to analyze the temperature, CO gas, and visibility to determine the available evacuation time, obtaining that a numerical fire simulation was performed to analyze the temperature, CO gas, and visibility to determine the available evacuation time. Therefore, this study proposes a method to derive the optimal coupled evacuation by combining the dynamic motion of smoke and the evacuation process. First, fire and smoke dispersion were simulated using Pyrosim software. Subsequently, evacuation simulations were performed using Anylogic numerical software. The available safe evacuation time for each evacuation path was obtained by analyzing the temperature, CO concentration, and visibility in the fire simulation results, and the evacuation times under different fire source locations, different personnel densities, escalator-coupled staircase evacuation, and staircase-only evacuation were determined from the computational results of the Anylogic software. Therefore, it is recommended that escalators be used as auxiliary evacuation staircases to improve evacuation efficiency.

## Fire scenario and personnel evacuation settings of the atrium shopping mall

### Building overview

**Fig 1:**
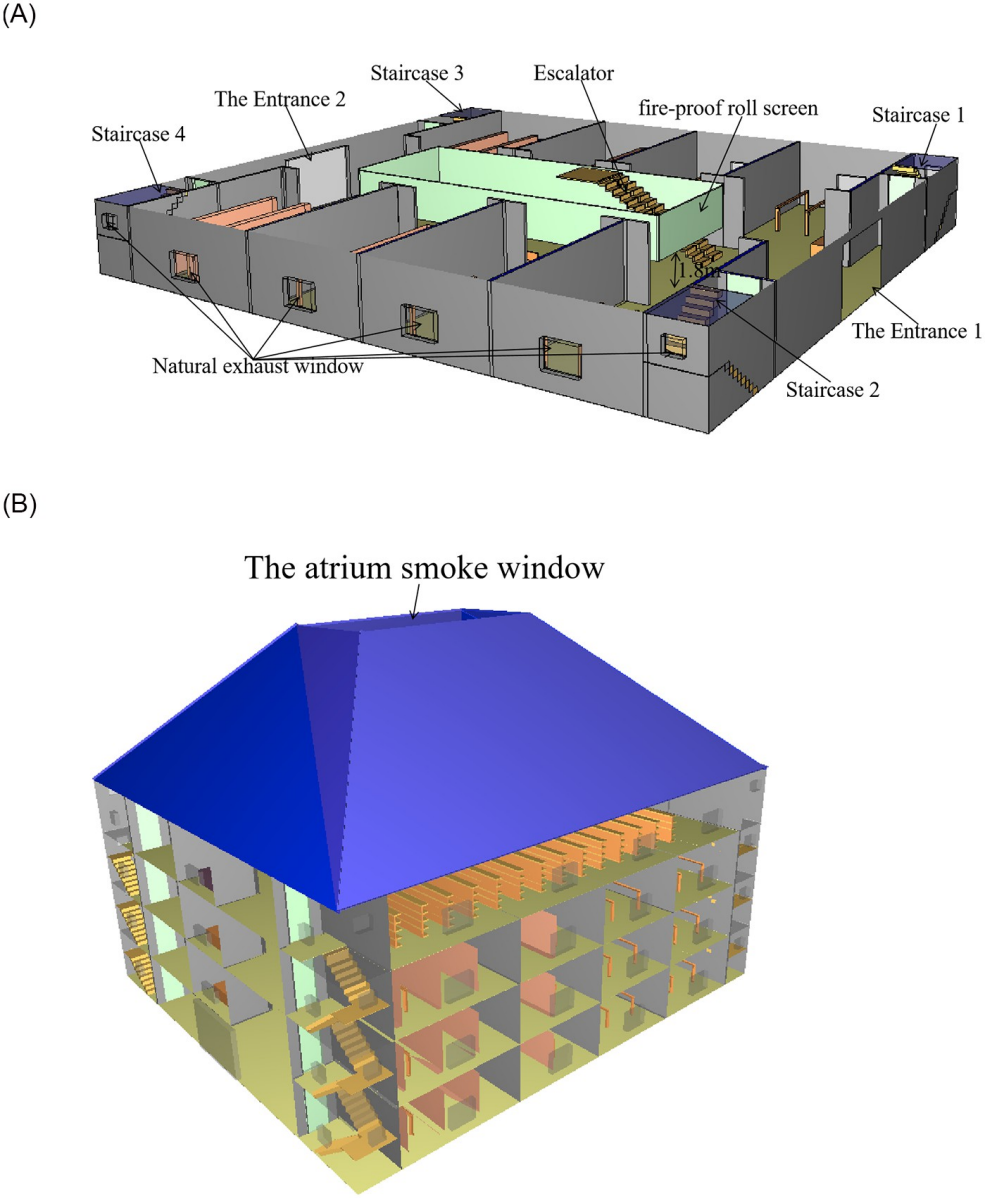
The 1st floor and mall model drawing. (a) The 1st floor model. (b) The atrium mall diorama.

The atrium mall consists of four floors. At its center, there is a rectangular atrium measuring 8 m × 20 m. On the first, second, and third floors, there are a total of eight stores, each with an area of 96 m^2^. The fourth floor, furthermore, contains five stores. Each floor has a height of 4.5 m. To facilitate evacuation, the mall is equipped with four evacuation staircases situated at the four corners of each floor. These staircases have a width of 1.5 m and provide direct access to the outside of the mall. And the width of the escalator is 0.8 m. Additionally, there are two evacuation exits on the first floor, which measure 3 m in width. These exits serve as the main entrances to the mall. To ensure safety, the mall is outfitted with automatic fire alarm systems, automatic sprinklers, and smoke extraction facilities. Fig 1 illustrates the layout of the first floor of the mall and provides an overview of the overall model.

### Parameter setting for fire scenarios

**Fig 2:**
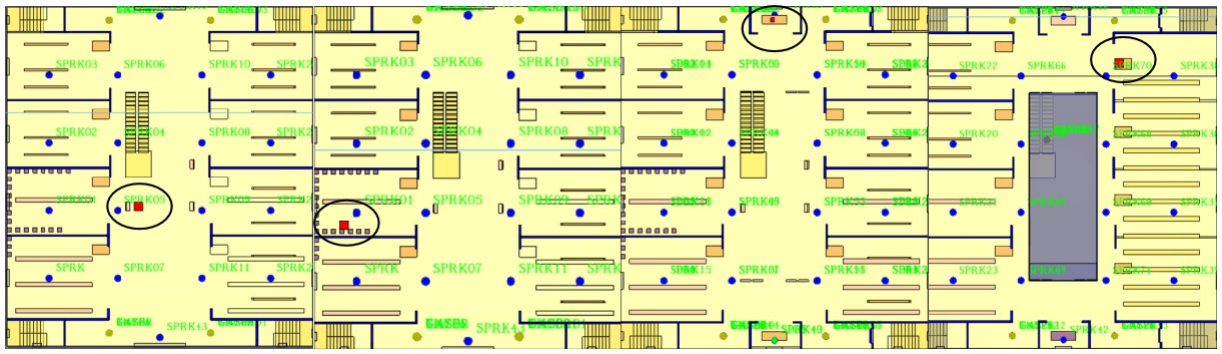
Fire source location map for each fire conditions. (a) The case 1. (b) The case 2. (c) The case 3. (d) The case 4.

Taking into account the Technical Guidelines for Smoke Prevention and Smoke Exhaust Systems in Buildings (GB51251-2017), the fire size is determined to be 3 MW in scenarios where the automatic sprinkler system is operational. Therefore, in this study, the fire source power is set at 3 MW, and the fire growth type is determined to be "fast t^2^" growth type. Given the presence of numerous wooden shelves, tables, chairs, and decoration materials within the mall, the fire growth coefficient is set to 0.044 kW/s^2^, and the fire response is defined as "WOOD___PINE." The simulation of the mall employs a grid size of 0.5 m × 0.5 m × 0.5 m, resulting in a total of 345,600 grid cells. And the total simulation time is 500 s. In this simulation, four fire source locations are set, which are respectively set in the middle hall on the first floor, the clothing store on the first floor, the utility room on the second floor and the supermarket on the fourth floor. The settings for the atrium was shown in Table 1; the fire source locations were shown in Fig 2. Both the atrium and the stores employ natural smoke exhaust systems. According to GB14102-2005, the descending speed of vertical roll fire shutter is 2 m/min-7.5 m/min. In this paper, the descending speed of the fire shutter is 5 m/min. So the time needed for the fire shutter to fall to 1.8m from the ground is 33s. The status of the fire shutter is illustrated in Fig 1A.

**Table 1:** Fire condition setting.

Fire conditions	The fire location	The fire size class/MW
**Case 1**	1st floor atrium	3
**Case 2**	The clothing store on the first floor	3
**Case 3**	Utility room on the second floor	3
**Case 4**	The supermarket on the fourth floor	3

### Fire hazard criteria

Smoke inhalation poses a significant risk to individuals during building fires [16, 17]. As such, it is crucial to consider the effects of smoke on human safety when assessing fire incidents. Key factors to consider include the height of the smoke layer at a distance of 2.0 m from the ground, temperature, and visibility. In the present study, we define the critical values for temperature and carbon monoxide (CO) concentration during evacuation as 60°C and 2.5×10–4 kg∙m^-3^, respectively. Because the mall is a large space, the critical value for visibility is 10.0m [18, 19].

### Personnel evacuation setting

#### Introduction to Anylogic software

Anylogic software, developed by XJ Technologies, is a simulation tool utilized for conducting evacuation simulations using the social force model. It offers three distinct simulation modeling methodologies: multi-agent, discrete event, and system dynamics. Notably, Anylogic software stands out as relatively novel pedestrian simulation software that bears greater resemblance to real-life pedestrian characteristics compared to conventional simulation software. It facilitates visual tracking of the flow of individuals through a facility, enabling real-time updates of population density and distribution at any given moment.

#### The atrium shopping mall environment modeling and pedestrian flowchart construction

**Fig 3:**
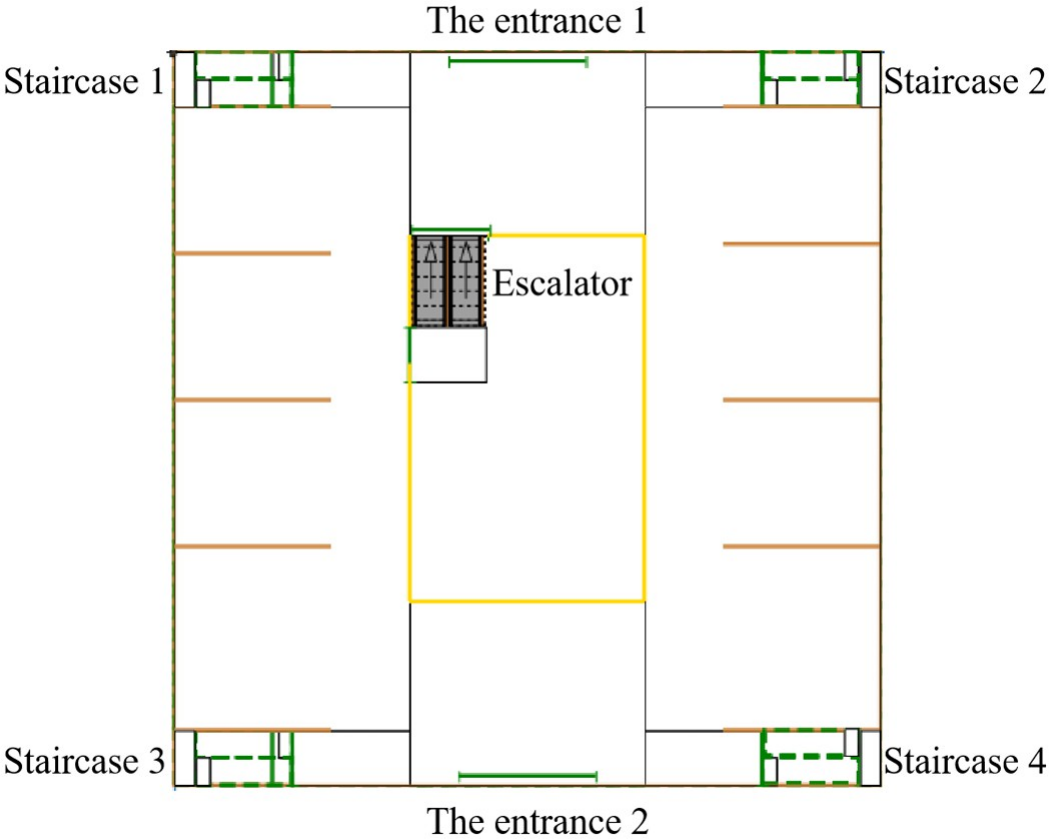
A two-dimensional physical model of an atrium shopping mall.

The Anylogic software’s pedestrian library incorporates various parameters, such as walls, rectangular areas, target lines, and escalator groups, among others [20]. These components enable the creation of individual floors within the mall, allowing for the depiction of level layers. The software can draw the outer perimeter and inner walls of the mall using wall elements and designate evacuation exits with target lines. Additionally, rectangle areas and rectangles are utilized to construct stairs, while escalator groups facilitate the representation of escalators within the two-dimensional physical model, as illustrated in Fig 3.

**Fig 4:**
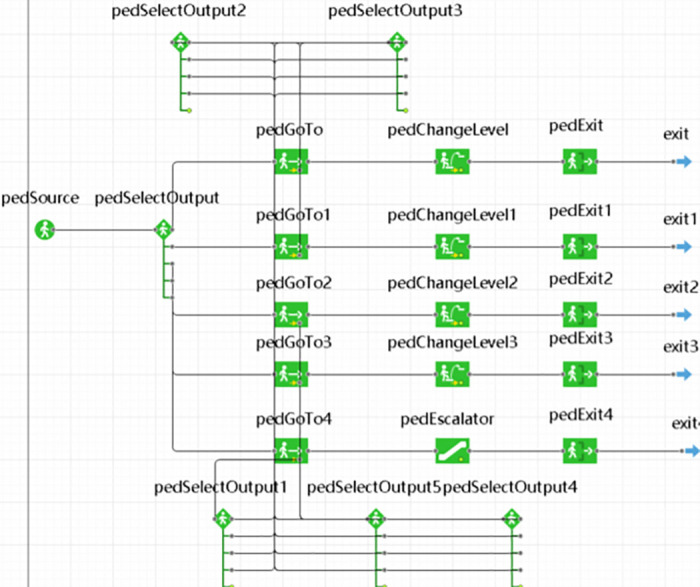
Evacuation logic diagram of 4th floor.

Based on the established model of the environment, the evacuation process for individuals is formulated using the pedestrian library and process library. The PedSelectOutput feature is configured to prioritize the selection of the nearest staircase or escalator by individuals. Events are programmed such that when the evacuation staircase or escalator on each level reaches the designated safe evacuation time, individuals are restricted from passing and are redirected to select closer alternative exit routes [21]. During the simulation runtime, the visual data window captures and logs the number of evacuees and the duration of the evacuation process. Fig 4 presents a partial display of the implemented logic.

#### Basic parameter setting

In accordance with building regulations for the atrium mall business hall, the personnel occupancy is determined to fall within the range of 0.3–0.6 people∙m^-2^. Therefore, this study conducted simulations for four different densities: 0.3, 0.4, 0.5, and 0.6 people∙m^-2^, in order to evaluate the evacuation times under each scenario. Furthermore, the direction of motion for the escalators in this study was set to be downward. The basic parameters for the personnel were derived from statistical data obtained from the mall, and are summarized in Table 2.

**Table 2:** Parameters of mall personnel.

Personnel category	Children	Females	Males	Elderly
**evacuation speed/(m/s)**	1.1	1.3	1.33	0.9
**shoulder width/cm**	27	38.7	41.5	30.8
**proportion/%**	10	40	30	20

## Simulation results and analysis

### Simulation analysis of smoke in the atrium shopping mall

Pyrosim software was used to simulate the smoke transport and changes in smoke temperature, CO concentration and visibility of an atrium mall when the fire shutter was lowered to the 1.8 m position under different fire conditions. The simulation results were used to compare and analyze the impact of each fire source location on the evacuation of people.

### Analysis of flue gas migration

**Fig 5:**
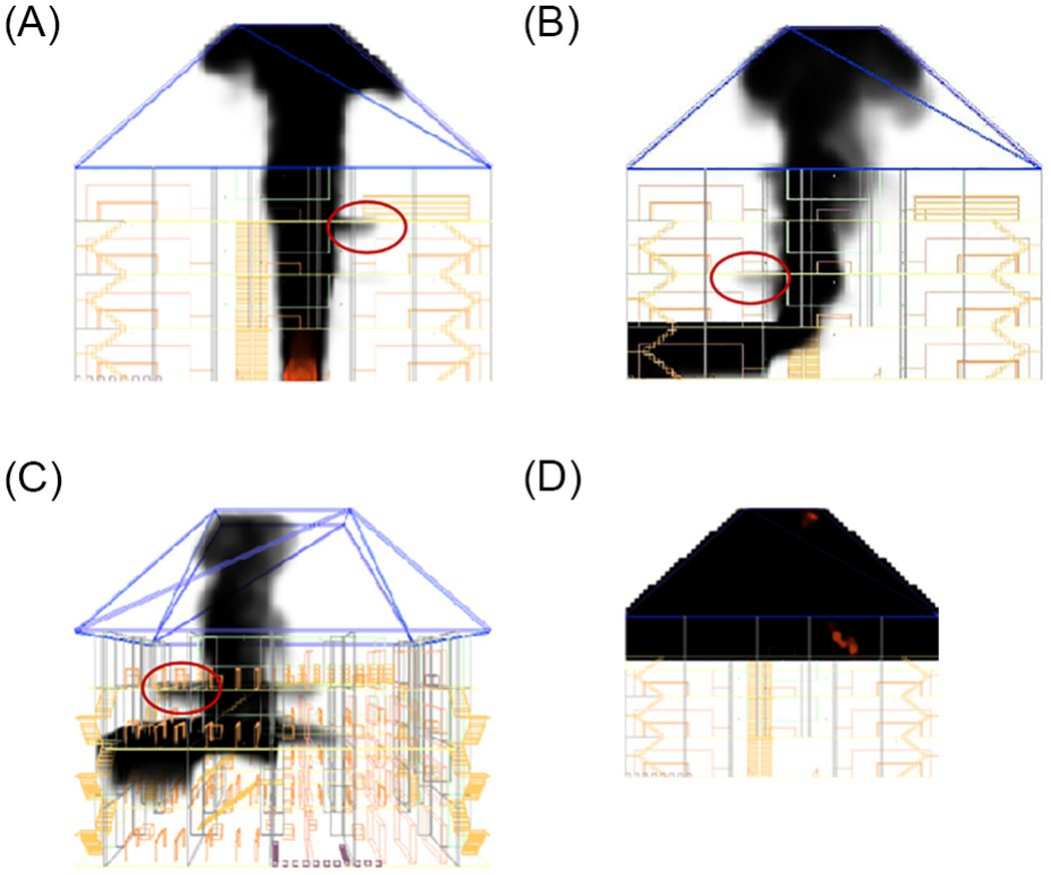
Flue gas migration diagram under various working conditions. (a) The case 1 at 97.8 s. (b) The case 2 at 120 s. (c) The case 3 at 80 s. (d) The case 4 at 500 s.

When a fire occurs, fire shutters don’t have to come down where the fire source is located floor. And the fire shutters on other floors are lowered to 1.8m from the ground. From the Fig 5A, it can be seen that the smoke of the case 1 first moves to the top of the atrium, and then spreads around. When the other floors fire shutters fall to 1.8m, the smoke is the first to spread to the 3rd floor corridor through the space reserved for the 3rd floor fire shutters at 97.8 s. As can be seen in Fig 5B, in Case 2 the smoke is transported towards the roof, then outwards along the top plate and subsequently towards the top of the atrium in the atrium area. At 120 s, the smoke will be through the 2nd floor fire shutter reserved gap to the 2nd floor corridor. From the Fig 5C, at the beginning of the fire, smoke transport of the case 3 is similarly with the case 2. And the smoke in 80 s through the reserved gap to the third floor. From Fig 5D, it can be concluded that in the case 4, at 500 s, the smoke only moves in the top floor space area and has little effect on other floors.

The results of the analysis showed that in Case 4, when the fire shutter was left at 1.8 meters, there was almost no smoke dispersion to the other floors during the simulation time. However, the smoke from Case 3 spread to other floors first through the gap reserved for the fire shutter, followed by Case 1 and finally Case 2. The time for the smoke to spread to other floors through the reserved gap was 80s, 97.8s and 120s, respectively.

### Temperature analysis

According to the temperature slices set at 1.8 m on each floor, it is known that the temperature reaches up to 200°C, but only exists near the fire source. And there are fewer areas where the temperature is above the evacuation critical temperature in the stores and atrium area where the fire source is located, so the influence of temperature on the evacuation of people is mainly in the vicinity of the fire source.

### Analysis of CO concentration

**Fig 6:**
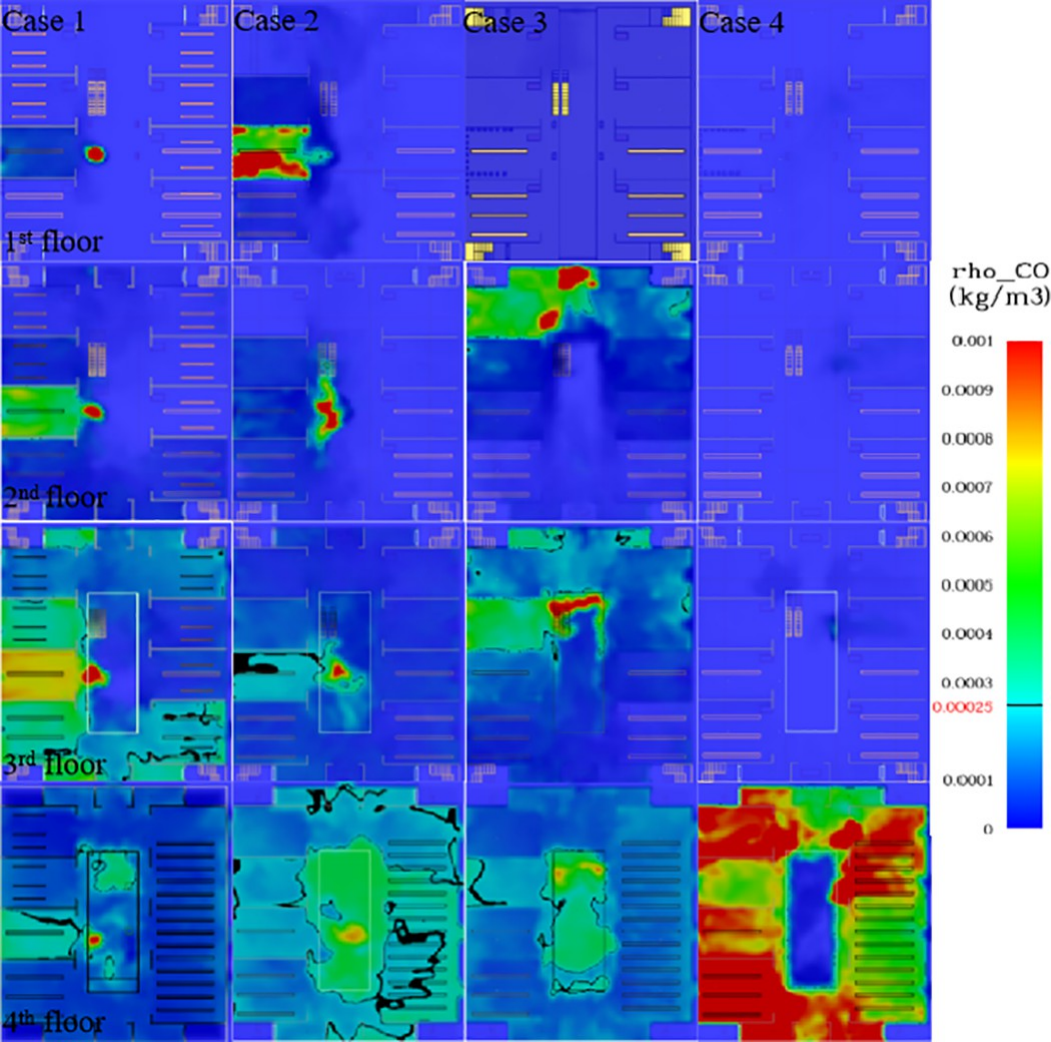
Cloud map of CO concentration in each working condition at 500 s.

Slices of CO concentration at 2 m above the ground for each case are shown in Fig 6. As can be seen from the figure, the CO concentration in Case 1 has the greatest impact on the stores adjacent to the fire source. Compared to other floors, the impact of CO concentration on evacuation needs to be considered for most of the 3rd floor when the fire source is located in the atrium of the first floor, followed by the top floor. Therefore, the evacuation staircases on the 3rd floor and the escalator on the 4th floor need to take into account the impact of CO concentration on evacuation. In case 2, the degree of influence of CO concentration on evacuation is, in descending order, 4F, 3F, 1F and 2F. The effect of CO concentration on evacuation is mainly considered for each evacuation staircase on the 4th floor. According to the slices of CO concentration on each floor of Case 3, the 1st floor is not affected by the smoke concentration. the 2nd floor has a higher CO concentration in the stores near the fire source and in Staircase 1. the 3rd floor has a similar CO concentration as the 2nd floor, and the 4th floor has a higher CO concentration at the atrium. Hence, CO concentration is considered for evacuation staircases 1 and 2 on the 2nd and 3rd floors, and for escalators on the 3rd and 4th floors. The CO concentration in Case 4 affects only the 4th floor, and each evacuation staircase needs to consider the effect of CO concentration on personnel evacuation.

### Visibility analysis

**Fig 7:**
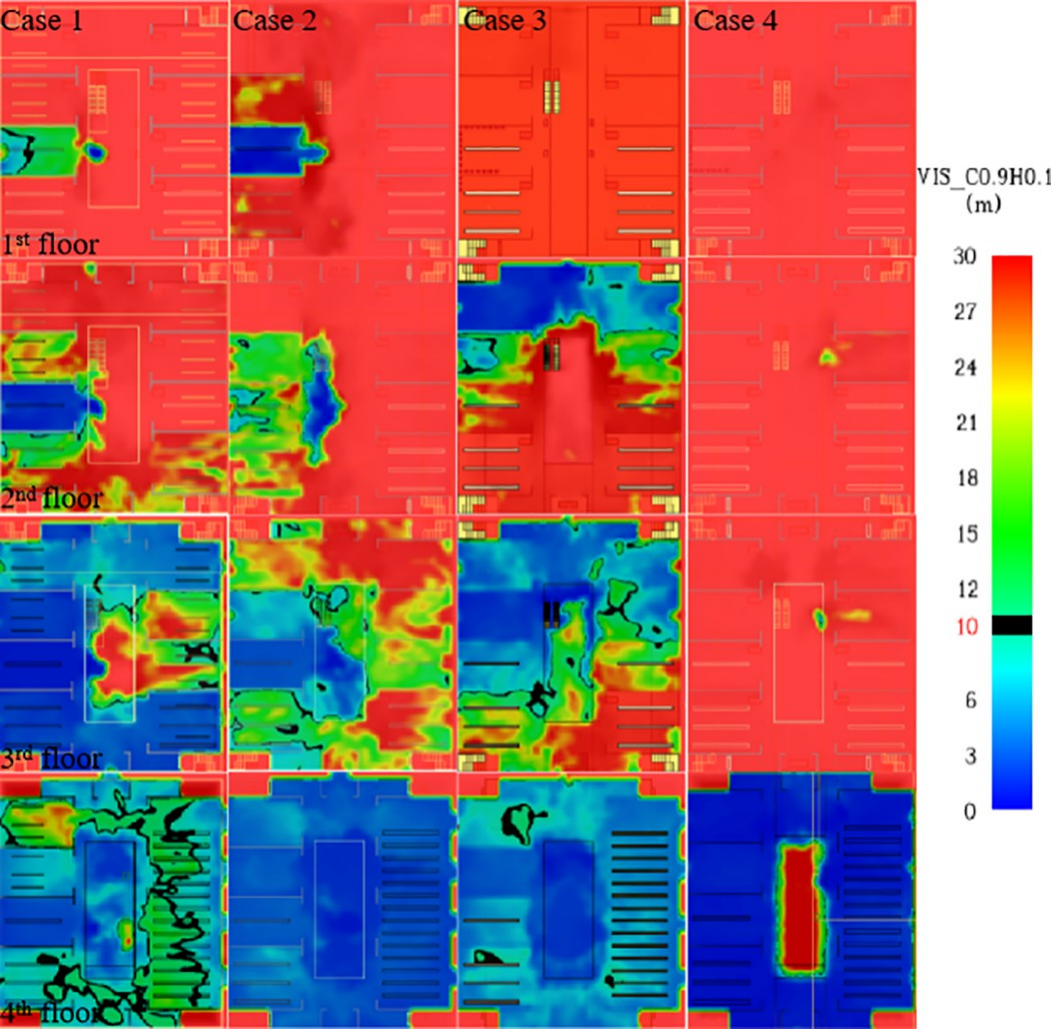
Visibility cloud image at 500 s for each working condition.

Fig 7 shows the distribution of visibility at 500s at 2 meters height on each floor under different working conditions. When the fire source is set at different floors, the visibility at level 1 is the least affected, while the most significant effect is at level 4. By analysing the visibility cloud at the escalator, under Case 2, the visibility at the 2nd floor was lower than the critical value; while under Case 1, 2 and 3, the visibility at the 3rd floor was also lower than the critical value; moreover, the visibility at the escalator at the 4th floor did not reach the safety standard under all the cases. For visibility at the evacuation staircases on each floor, in Case 1, the visibility on the 3rd and 4th floors was insufficient to meet the safety evacuation requirements. For Case 2, the visibility at staircases 1 and 3 on the 3rd floor and all evacuation routes on the 4th floor were below the critical values. For Case 3, the visibility of evacuation staircases 1 and 2 on the 2nd and 3rd floors and all evacuation staircases on the 4th floor were also below the safety criteria. Finally, in Case 4, only the visibility of the 4th floor evacuation staircase is of concern for its potential impact on the evacuation process.

**Fig 8:**
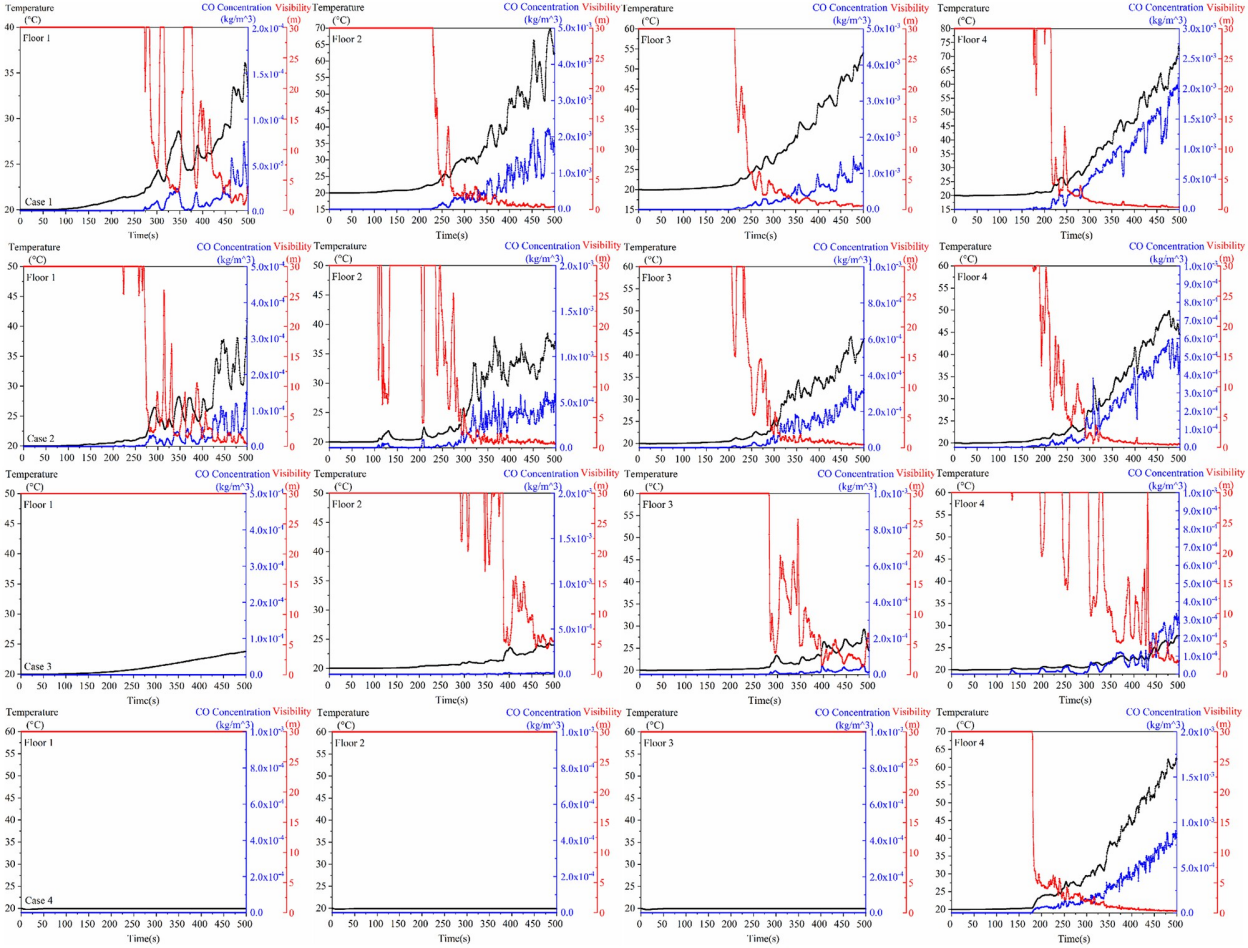
Temperature, CO concentration, visibility at escalator for each case.

When analysing the effects of different fire source locations on smoke transport, temperature, carbon monoxide (CO) concentration and visibility when the escalator fire shutter door is lowered to 1.8m, the third and fourth floors of the building are particularly affected by smoke when the fire source is located in the atrium area of the ground floor of the building. If the fire source is located in the commercial area of the first and first floors, the top floors are more severely affected by smoke. However, when the fire source is on the top floor of the building, the other floors are virtually unaffected by smoke, and at this point the escalator has the highest evacuation safety factor of all scenarios. In order to gain a deeper understanding of the specific effects of fire smoke on the escalators on each floor, we conducted a comparative analysis of the temperature, visibility and CO concentration at the entrance of the escalator in each scenario, as shown in Fig 8.

As can be seen from the graph in Fig 8, visibility has the most significant effect on evacuation. When the fire source was located on the first floor, the smoke affected the evacuation at the escalators on all floors. The effect of smoke on the escalator is most significant in Case 1, where the temperature at the escalator reaches a maximum of 75°C. The available evacuation times for all floors are determined by the visibility and are 294s, 241s, 241s, and 216s, respectively. the temperature at the escalator does not affect evacuation in Case 2, and the evacuation times for all floors are determined by the visibility 274s, 118s, 250s, and 212s. the temperature at the escalator in Case When the fire source of Case 3 is located on the 2nd floor, the 1st floor is not affected by smoke, and the available evacuation time of the escalators on each floor is 500s, 387s, 284s, and 305s. The fire source of Case 4 is located on the top floor, and the escalators on the other floors do not need to take into account the effect of smoke, and the available safety time of the escalators on the 4th floor is 182s.

In order to obtain the available safe time for evacuation staircase for each condition. The temperature, visibility and CO concentration of each floor were analyzed and summarized, and the available safe evacuation time for each fire condition was obtained as shown in Table 3.

**Table 3:** Evacuation stairs and escalators available safe evacuation time.

Fire conditions	Floor	Available safe evacuation time (s)				
		Staircase 1	Staircase 2	Staircase 3	Staircase 4	Escalator
**Case 1**	1^st^ floor	500	500	500	500	294
	2^nd^ floor	500	500	449	500	241
	3^rd^ floor	351	415	204	363	241
	4^th^ floor	471	500	500	411	216
**Case 2**	1^st^ floor	500	500	500	500	274
	2^nd^ floor	500	500	500	500	118
	3^rd^ floor	450	500	460	500	250
	4^th^ floor	331	348	368	340	212
**Case 3**	1^st^ floor	500	500	500	500	500
	2^nd^ floor	120	192	500	500	387
	3^rd^ floor	133	231	500	500	284
	4^th^ floor	414	405	423	420	305
**Case 4**	1^st^ floor	500	500	500	500	500
	2^nd^ floor	500	500	500	500	500
	3^rd^ floor	500	500	500	500	500
	4^th^ floor	111	87	182	143	182

### Simulation analysis of personnel safety evacuation

After a fire, people need to evacuate to the safe area in the shortest time, and the safe passage affects the evacuation time of people [13]. By analyzing the above fire conditions, it can be seen from Table 3 that escalators are not always used as evacuation stairs under fire conditions. Comparing the four working conditions, the escalator is used for the longest time in case 4, and all floors are not affected by smoke except the fire source floor.

**Fig 9:**
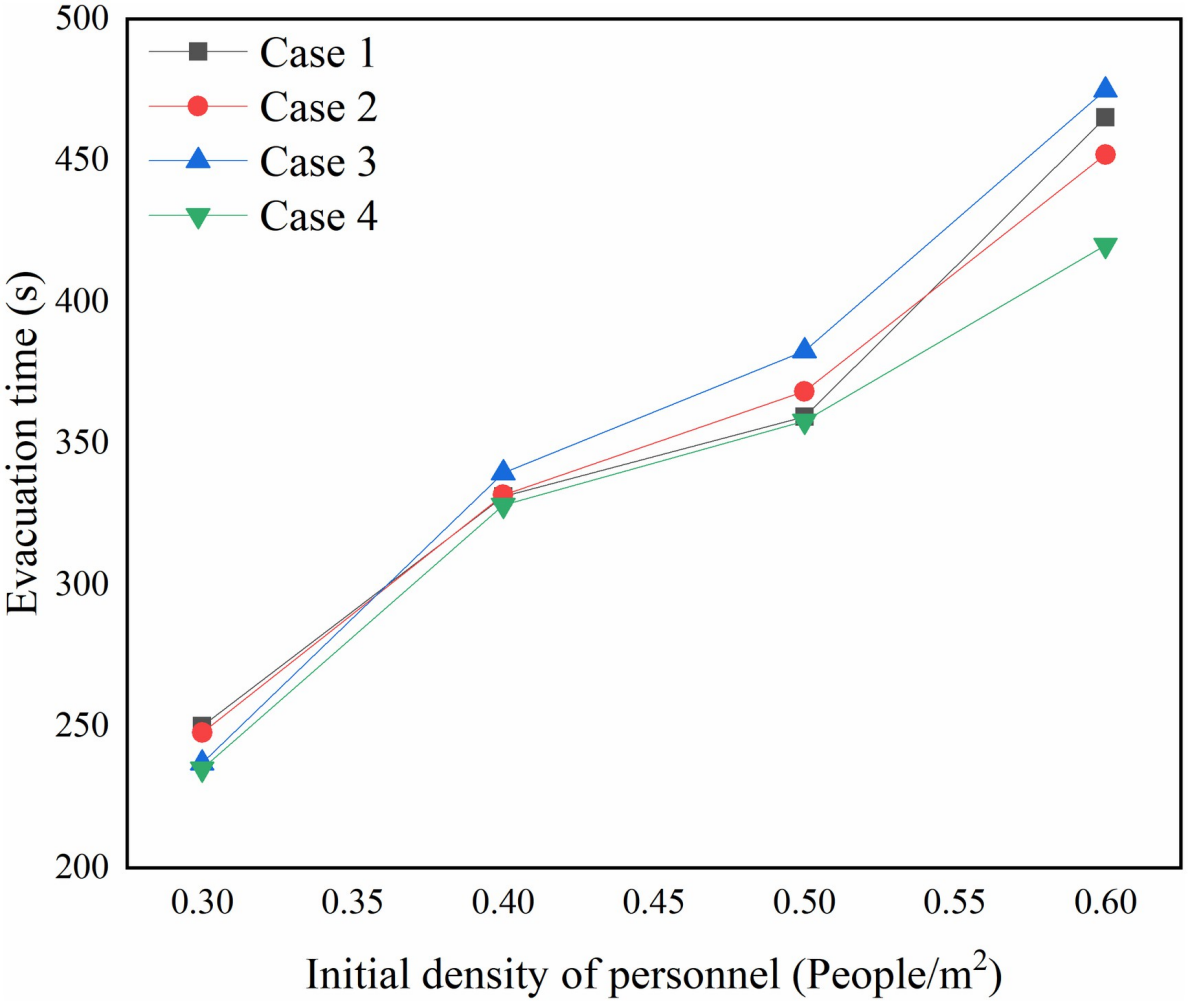
Evacuation time curve with personnel density.

According to the evacuation time of each working condition under different initial density of personnel recorded in the data window, the scatter plot was drawn by Origin software, and the curve of each working condition was fitted according to the change trend of the scatter plot, and the fitted curve is shown in Fig 9.

As can be seen from Fig 9, the evacuation time under the four working conditions grows with the increase of the initial density of people, and the difference of the evacuation time under the coupling of escalator and staircase is smaller when the density of people is less than 0.4 people∙m^-2^. When the personnel density is greater than 0.4 people∙m^-2^, the evacuation time of working condition 3 is greater than other working conditions. And the evacuation time of case 4 is the least under different personnel densities.

**Fig 10:**
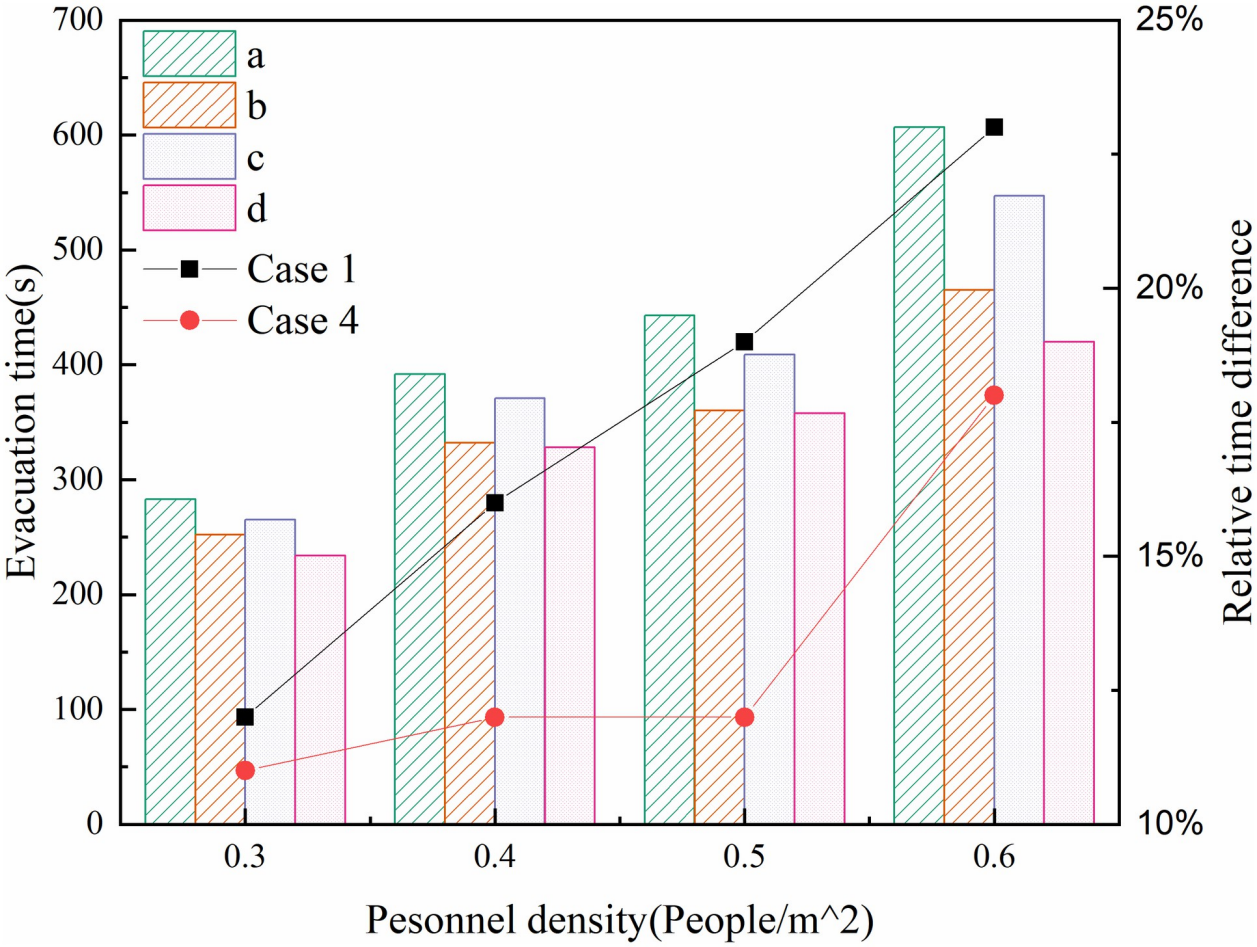
Evacuation time under different evacuation modes in case 1 and 4. Where, Where, a and b respectively represent stairway evacuation and escalator stairway coupling evacuation in case 1; c and d respectively represent stairway evacuation and escalator stairway coupling evacuation in case 4.

To study the effect of escalator on evacuation time. Taking working conditions 1 and 4 as examples, the evacuation time curves of evacuation with or without escalator in the two working conditions were obtained, as shown in Fig 10. As can be seen from the figure, the evacuation time for both cases under coupled evacuation is smaller than that of evacuation by stairs only. When the initial density is 0.3, 0.4, 0.5 and 0.6 persons∙m^-2^, the shortened evacuation time is 33.8s, 61.8s, 84s and 141.9s for case 1 and 30 s, 43.8 s, 51 s and 96.9 s for case 2, respectively, and when the initial density is 0.6 people∙m^-2^, the coupled evacuation time of case 1 is 23% shorter than that of stairs only.

**Fig 11:**
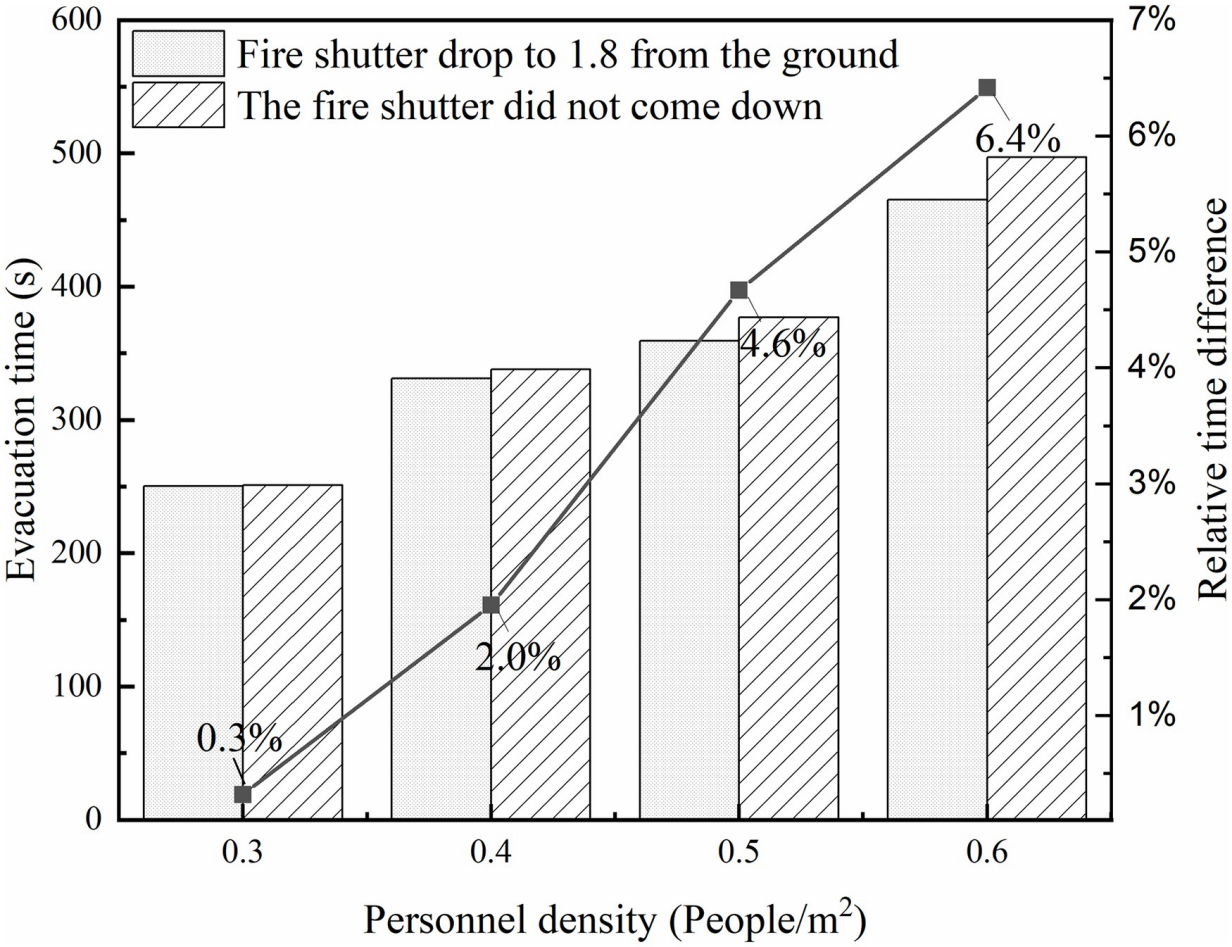
Evacuation time of different states of fire shutters in working condition 1.

In order to study the influence of the fire shutter at the escalator falling to 1.8m from the ground or not falling on the evacuation of personnel, so the evacuation time of the fire shutter not falling and the fire shutter falling to 1.8m from the ground in working condition 1 is shown in Fig 11. According to the figure, under different personnel density, the evacuation time of the fire shutter falling to 1.8m from the ground is smaller than that of the fire shutter not falling, and as the personnel density increases, the difference between the evacuation time of the two states is larger. At 0.6 persons∙m^-2^, the evacuation efficiency of the fire shutter descending to 1.8m from the ground is 6.4% higher than that of the undescended fire shutter.

## Discussion

The objective of the current study is to analyze the effect of evacuation using escalators on evacuation of people under different fire source locations. Through the numerical simulation of fire development and evacuation, when the fire source is located in the atrium of the first floor and the fire shutter is lowered to 1.8m from the ground, the smoke has a more significant effect on the 3rd floor compared to the top floor. Moreover, when the fire source is located in the top floor, the smoke has almost no effect on other floors. By analyzing the coupled evacuation time of stairs and escalators under each fire condition, it is obtained that the evacuation time is the shortest when the fire source is located on the top floor, and the evacuation time is the longest when the fire source is located in the sundry room on the 2nd floor. And the coupled evacuation time can be reduced by up to 23% than only the staircase evacuation time. The evacuation efficiency can be increased by 6.4% when the fire shutter is lowered to 1.8m than when the fire shutter is not lowered. This study can provide a new evacuation mode for buildings with escalators.

It was suggested that when the fire source was located in the middle of the atrium on the first floor, the smoke spread most widely and had the greatest impact on the top floor corridor (Zhang, 2020). This does not appear to be the case. This is mainly due to the fact that in this paper, the fire shutter of the atrium building was not fully lowered, but only lowered to 1.8m from the ground level, the smoke will spread to other floors through the gap. According to Fig 5, it can be seen that at 97.8s, the smoke spreads to the 3rd floor due to the gap in the fire shutter. This led to the worst impact of smoke on the 3rd floor when the fire source was located in the atrium of the first floor. Moreover, the critical time required for fire to become a hazard determined in terms of visibility was significantly shorter than temperature or CO mass fraction(Yi, 2019). This finding can also be demonstrated by the Fig 8. In addition, according to Fig 8, it can be seen that escalators can assist in the safe evacuation of people within a certain period of time, especially when the source of the fire is located on the top floor, the escalators at other floors are less affected by the smoke, and the use of escalators in the process of evacuation has the longest available safe evacuation time. The paper analyzes the evacuation of people in working conditions and concludes that the longest evacuation time was found when the fire source was located in the middle level, a finding that is consistent with a recent paper (Song, 2020). According to previous studies, some scholars have obtained that escalator-coupled evacuation of stairs results in less personnel evacuation time, and the time for coupled evacuation is obtained to be half of that for staircase-only evacuation (Hiroyuk et al., 2012; Tang, 2020; Ma, 2021). In this paper, we similarly obtained that escalator-coupled evacuation stairs can take less time for personnel evacuation. And the coupled evacuation of personnel under fire scenario is added. The maximum coupled evacuation time is obtained to be 24% less. Mainly due to the influence of fire, the escalator can not always act as an evacuation staircase, so the evacuation time is not reduced by half. Additionally, the evacuation situation when the fire shutter was lowered to 1.8m from the ground and when the fire shutter was not lowered were compared and analyzed. It was obtained that the fire shutter descending to 1.8m from the ground is more favorable to the evacuation of people using escalator.

However, the limitation of this study is that it does not take into account the variation of evacuation speeds on escalators, and it does not consider the issue of people loading at escalators. In conjunction with the case study, several knowledge gaps in the area of escalators are specific areas that require further investigation including (1) the speed of evacuating people in different states of an escalator through field testing. (2) How escalators should be lowered to a height of 1.8m above the ground. (3) Safety guides should be installed at escalators to prevent and control overloading of people at escalators.

## Conclusions

In this paper, Pyrosim software is used to simulate the smoke spread when the fire shutter at the escalator drops to 1.8m from the ground under different fire source locations, and the available safe evacuation time under fire conditions is applied to the Anylogic software for personnel evacuation simulation, and the following conclusions are mainly drawn.

1. When the fire source is located on the top floor, the smoke has almost no effect on other floors. And the escalator coupled stairs can shorten the evacuation time.
2. Escalators coupled with evacuation stairs can improve the evacuation efficiency, and the coupled evacuation time can be shortened by 23.37% compared with the evacuation time of stairs only. And when the fire shutter at the escalator is lowered to 1.8m from the ground, the evacuation time is 6.41% shorter than when the fire shutter is not lowered.
3. In order to evacuate the escalator more safely, we can consider adding a water curtain at the fire shutter and setting up an escalator for voice announcement of evacuation.

## Supporting information

S1 Data (XLSX)

S2 Data (PDF)

S3 Data (XLSX)

## References

[1] Wang C., Song Y. Fire Evacuation in metro stations: modeling research on the effects of two key parameters[J]. Sustainability,2020,12(2):684–695. doi: 10.3390/su12020684

[2] Kinsey, M.-J., Galea, E.-R. and Lawrence, P.-J. Lawrence. Extended model of pedestrian escalator behaviour based on data collected within a Chinese underground station. In Proceedings of the Human Behaviour in Fire Conference, 2009, 173–182.

[3] Ma Z.-C., Xiao Z.-N., Chen J., Zheng C.-C, Xu W.-J. Study on smoke and evacuation in atrium island shopping mall[J]. Fire Science and Technology, 2019, 38(01):86–89.

[4] Huang Z. The auxiliary role of escalators and moving sidewalks in safe evacuation[J]. Journal of Nanchang College, 2010, 25(03):167–168.

[5] NAOKO K., HASEMI Y., MORIYAMA S. Feasibility of upward evacuation by escalator—an experimental study[J]. Fire & Materials, 2012, 36(S1):429–440. doi: 10.1002/fam.1118

[6] HIROYUKI K., SEKIZAWA A., TAKAHASHI W. Study on availability and issues of evacuation using stopped escalators in a subway station[J]. Fire & Materials, 2012, 36(S1):416–428. doi: 10.1002/fam.1097

[7] Tang J.- L. Study on fire evacuation optimization Planning of large commercial buildings[D]. MA.Eng. Thesis, The University of Dalian University of Technology, 2020.

[8] Ma H., Zhang H.-B., Zhu H.-J. Study on multi-channel coupling evacuation strategy of stairs, escalators and elevators in commercial complex[J]. Journal of Safety Science and Technology, 2021, 17(03):149–154.

[9] Xie J.-B., Chen K.-C., Trevor H. Kwan, Yao Q.-H. Numerical simulation of the fire emergency evacuation for a metro platform accident[J]. SIMULATION, 2021, 97(1):19–32.

[10] Guan C., Zhang Z.-Z, Li J, WU B, Yan Q.-H. Analysis on cause factors and evolution paths of fire accidents in buildings. China Safety Science Journal, 2022, 32(4),163–170.

[11] Huang X., Jin J., Lin Z., Che L., Liu J. Dynamic evacuation path planning for fire disaster of deep underground space based on A* algorithm. Journal of Beijing University of Technology. 2021, 47(7):702–709.

[12] Wang F., Zhang Y., Ding S., Huang X. Optimizing phased-evacuation strategy for high-rise buildings in fire. Journal of Building Engineering. 2024, 95:110084.

[13] Lotfi N., Behnam B., Peyman F. A BIM-based framework for evacuation assessment of high-rise buildings under post-earthquake fires[J]. Journal of Building Engineering, 2021, 43: 102559.

[14] Yan Z., Wang Y., Chao L.-X., Guo J. Study on Evacuation Strategy of Commercial High-Rise Building under Fire Based on FDS and Pathfinder[J]. CMES-Computer Modeling in Engineering & Sciences, 2024, 138(2):1078.

[15] Cui Y., Wang H., You B., Cheng C., Li M. Simulation Study on Fire Product Movement Law and Evacuation in a University High-Rise Teaching Building. Applied Sciences. 2023, 13(18): 10532.

[16] Ye C.-H., Liu Y.-C., Sun C., Jiang Y.-Q., Wang B. Simulation study on personnel evacuation considering impacts of fire products[J]. China Safety Science Journal, 2020, 30(06):142–151.

[17] Lv W., Wang J.-H., Li J.-W., Chen W.-T., Fang Z.-M. Analysis of evacuation risk of wide-body aircraft in fire scene[J]. China Safety Science Journal, 2023, 33(03):141–146.

[18] Guo S.-C. Studies on personnel available evacuation time TASET of large shopping mall fire[D]. The University of Xi’an University of Architecture and Technology, 2016.

[19] Wang J.-Y., Zong R.-W., Lu S.-X. Dynamic evaluation of consequences of toxic gas dispersion in fire of crowded places[J]. China Safety Science Journal, 2021, 31(12):167–175.

[20] Ma W.-W., Wang J, Wu Q.-M., Guo X.-H. Guides setting and analogue simulation in emergency evacuation[J]. Journal of Safety and Environment, 2017, 17(02):625–629.

[21] Zhou W.-K., Zhao H., Zhou H.-J. The research of University dormitory fire evacuation based on AnyLogic simulation[J]. Fire Science and Technology, 2014, 33(12):1383–1386.

