# Supplementary material for: Research on coupling evacuation of escalator and staircase in fire scenario
Source: PLoS One. 2025 Feb 4;20(2):e0314455. doi: 10.1371/journal.pone.0314455 (PMC11793821; doi:10.1371/journal.pone.0314455)
Supplement: S2 Data — (PDF) [file pone.0314455.s002.pdf]

Fig. 9

Personnel density（people/m2）	case 1	case2
0.3	252	247
0.4	332	332
0.5	360	368
0.6	465	452
case 3	case 4	
238	234	
340	328	
382	358	
474	420	

Fig. 10

Personnel density（people/m2）	Evacuation time of case 1(Stairs only)	Evacuation time of case 1(Stair coupled escalator)
0.3	283	252
0.4	392	332
0.5	443	360
0.6	607	465
Evacuation time of case 4(Stairs only)	Evacuation time of case 4(Stair coupled escalator)	Relative evacuation time(case 1)
265	234	12.00%
371	328	16.00%
409	358	19.00%
547	420	23.00%
Relative evacuatitime(case 4)		
11.00%		
12.00%		
12.00%		
18.00%		

Fig. 11

Personnel density（people/m2）	Fire shutter lowered to 1.8m above the floor	Fire shutter not lowered
0.3	252	252.76
0.4	332	338.64
0.5	360	376.56
0.6	465	494.76
Relative evacuation time		
0.30%		
2.00%		
4.60%		
6.40%		
